# Doxycycline-Induced Antinuclear Antibody and Antineutrophil Cytoplasmic Antibody Associated Vasculitis: A Case Report and Literature Review

**DOI:** 10.1155/2020/3853671

**Published:** 2020-08-27

**Authors:** Davisson J. Wriston, Evan R. Norfolk, Lindsay M. Smith, Guoli Chen, David H. Bulbin

**Affiliations:** ^1^Department of Internal Medicine, Geisinger Medical Center, Danville, Pennsylvania, USA; ^2^Department of Nephrology, Geisinger Medical Center, Danville, Pennsylvania, USA; ^3^Department of Pathology, Geisinger Medical Center, Danville, Pennsylvania, USA; ^4^Department of Rheumatology, Geisinger Medical Center, Danville, Pennsylvania, USA

## Abstract

Drug-induced antineutrophil cytoplasmic antibody (ANCA)-associated vasculitis (AAV) has been increasingly recognized in the literature with numerous medications listed as causative agents in disease pathology. Doxycycline is a commonly prescribed medication within the United States which is a synthetic, broad-spectrum antibiotic with antimicrobial properties and at low doses exhibits anti-inflammatory effects. In this report, we describe a case of doxycycline-induced ANCA-associated vasculitis with laboratory and biopsy findings supporting the diagnosis, which to the best of our knowledge is the first described case of doxycycline-induced AAV in the literature. The patient was started on doxycycline for treatment of potential Lyme disease. She began to develop progressively worsening myasthenia, erythematous macular rash, anorexia, anemia, and fatigue for several weeks following the course of doxycycline with initial concern of a paraneoplastic process. Ultimately, the patient was discovered to be positive for antinuclear antibody (ANA), perinuclear antineutrophil cytoplasmic antibody (pANCA), and myeloperoxidase (MPO) antibody for which she was treated with a course of prednisone leading to complete remission of disease. A brief review of the pathogenesis of ANCA vasculitides will also be discussed within this article.

## 1. Introduction

Drug-induced antineutrophil cytoplasmic antibody (ANCA)-associated vasculitis is a unique subset of the ANCA-associated vasculitides which are classified as small vessel vasculitides. Small vessel vasculitis was divided into ANCA-associated vasculitis (AAV) and immune complex vasculitis in the 2012 revised Chapel Hill Consensus Conference. The division was made due to a higher level of confidence that ANCAs are directly involved in the pathogenesis of AAV which has been confirmed in follow-up literature [1, 2]. ANCA-associated vasculitis is a disease entity comprising microscopic polyangiitis (MPA), granulomatosis with polyangiitis (GPA), and eosinophilic granulomatous with polyangiitis (EGPA) that are all ANCA positive. There are several antigens to ANCA that have been discovered; however, there are two in particular that are part of diagnostic criteria for MPA, GPA, and EGPA. These antigens are proteinase 3 (PR3) and myeloperoxidase (MPO) which are almost exclusively found in neutrophils and monocytes [3]. ANCA indirect immunofluorescence against PR3 results in a diffuse cytoplasmic (cANCA) staining pattern, whereas indirect immunofluorescence staining against MPO results in a perinuclear pattern (pANCA). A perinuclear ANCA pattern can be seen with several other proteins such as elastase, cathepsin, *β*-glucuronidase, or lactoferrin [4]. A cytoplasmic ANCA pattern is almost exclusively caused by PR3.

AAV pathogenesis is multifactorial, comprising genetic factors, epigenetic factors, and environmental factors [5]. Environmental factors specifically include drugs or infections. Drug-induced AAV pathogenesis can be based on the medication's direct effects such as sulfasalazine causing necroptosis or the metabolites of the drugs that accumulate within the neutrophil binding to specific proteins, thereby making the proteins immunogenic [6]. Pathogenic ANCAs cause excessive neutrophil activation which leads to the persistent formation of neutrophil extracellular traps (NETs). NETs are used to trap and kill bacteria. NETs likely occur at a stable rate of production and degradation under homeostatic conditions [7]. However, persistent formation of NETs ultimately leads to damage of small vessels. ANCA production is also caused by NETs which leads to an unrelenting vicious cycle of ongoing inflammation [5]. There is still significant information to learn in regard to the exact pathogenesis of drug-induced AAV as some medications including minocycline, which is a tetracycline in the same class as doxycycline, do not appear to directly induce NET formation or inhibit NET degradation raising the question if another mechanism is at work for causing the drug-induced AAV [8].

Drug-induced ANCA-associated vasculitis presents similarly to primary AAV with clinical and serological profiles [9]. However, there are certain characteristics that drug-induced AAV alone seems to display. Most data on the subject are from retrospective studies with antithyroid drug-induced AAV compared to primary AAV. These data appear to be consistent with case reports consisting of other medications causing AAV. Specifically, patients with antithyroid drug-induced AAV, when compared to primary AAV, more often have skin damage, positive ANA, and good prognosis with high likelihood of disease remission [9, 10]. Kidney involvement is less common in antithyroid drug-induced AAV compared to primary AAV [11].

Doxycycline is a synthetic, broad-spectrum antibiotic with antimicrobial properties and at low doses exhibits anti-inflammatory effects [12]. Doxycycline and minocycline are part of the class of antibiotics called tetracyclines. There have been several case reports of minocycline-induced systemic erythematous lupus (SLE) and minocycline-induced AAV over the last 20 years. A single case report of doxycycline-induced subacute cutaneous lupus erythematous was reported in 2011 found on a Medline literature search [13]. However, there have been no previous cases reports of doxycycline-induced AAV.

## 2. Case Presentation

A 56-year-old woman with medical history notable only for lower extremity deep vein thrombosis in her 20s while being on oral contraceptive pills, 35 pack-year former smoker initially presented to her general practitioner for evaluation of jaw trismus which occurred a couple of days after she found a tick on her body. It was the first week of May and the patient resided an area with a high incidence of Lyme disease; therefore, her general practitioner prescribed a 3-week prescription doxycycline for treatment of potential Lyme disease. Soon after beginning the doxycycline, she began to experience worsening conjunctivitis, myalgias, generalized muscle weakness, erythematous rash, fatigue, weight loss, and anorexia. Doxycycline was continued by her general practitioner until day 18. Ultimately, the patient's Lyme titers were negative. The patient continued to experience worsening symptoms of muscle weakness, anorexia associated with weight loss, and fatigue with spontaneous resolution of conjunctivitis. She was seen in the emergency department for evaluation of symptoms at which time laboratory investigations revealed anemia with a hemoglobin of 10.4 g/dL. A repeat Lyme test was performed that also resulted as negative.

The patient was discharged home from the emergency department; however, one week later she was admitted in mid-June with nausea in addition to the aforementioned symptoms. Workup revealed microcytic anemia, ferritin of 478 ng/mL, erythrocyte sedimentation rate (ESR) > 120mm/hr, and rheumatoid factor of 52 IU/mL. The patient had further decline in hemoglobin with a negative fecal occult blood test. Hemolytic investigations were unrevealing. Anti-mitochondrial antibody and anti-smooth muscle antibody were both negative. ANA titer, anti-CCP IgG antibody, and anti-cardiolipin antibodies were pending at the time of discharge. The patient was informed to follow-up with her general practitioner for concern of underlying autoimmune disease.

The patient was seen at the rheumatology clinic two weeks following hospital discharge which was five weeks since stopping her doxycycline. She had worsening arthralgia, rash, 12 kilograms of unintentional weight loss, proximal weakness limiting her ability to ambulate with bloodwork from previous hospitalization revealing positive ANA 1 : 40 titer, anti-cyclic citrullinated antibody negative, and positive anti-phospholipid antibodies. The patient was hospitalized directly from the clinic as there was concern for an underlying paraneoplastic process, but other differentials included systemic lupus erythematosus and dermatomyositis. The patient underwent gastroscopy and colonoscopy to investigate for possible gastrointestinal malignancy which was unremarkable. She also had an echocardiogram to explore for possible myxoma which was also unremarkable. Admission laboratory work was notable for ESR  > 120 mm/hr, CRP 237 mg/L, ANA titer of 1 : 2,560, pANCA positive with confirmatory MPO resulting as 69.3 Units, creatinine of 1.0, and urinalysis (UA) reporting moderate blood with 5–9 white blood cells (WBCs) and 30–49 red blood cells (RBCs). Nephrology was consulted about obtaining a kidney biopsy given kidney involvement would mean exposing her to significant immunosuppressive agents. Repeat serial UAs consistently resulted in 5–9 RBCs without dysmorphic RBCs. Kidney biopsy was deferred given low suspicion of glomerulonephritis. The patient was started on IV methylprednisolone 500 mg daily for 3 days and transitioned to oral prednisone 60 mg daily with significant improvement in symptoms. The working diagnosis at the time of discharge was microscopic polyangiitis. She was discharged on prednisone 60 mg daily with a slow taper, azathioprine 100 mg daily, and omeprazole as well as follow-up appointments with nephrology and rheumatology.

At her follow-up rheumatology visit one month after hospital discharge, she was noted to have new mildly pruritic, erythematous papular rash on her upper and lower extremities; however, her previous systemic symptoms of fatigue, anorexia, and myasthenia were resolving. The new rash was concerning for drug eruption from azathioprine versus leukocytoclastic vasculitis from MPA. She was able to be sent directly to the dermatology clinic for skin biopsy. The patient was continued on azathioprine and prednisone until biopsy results showed the rash to be consistent with drug eruption without any evidence of vasculitis. Azathioprine was discontinued, and the rash resolved without further intervention.

**Figure 1:**
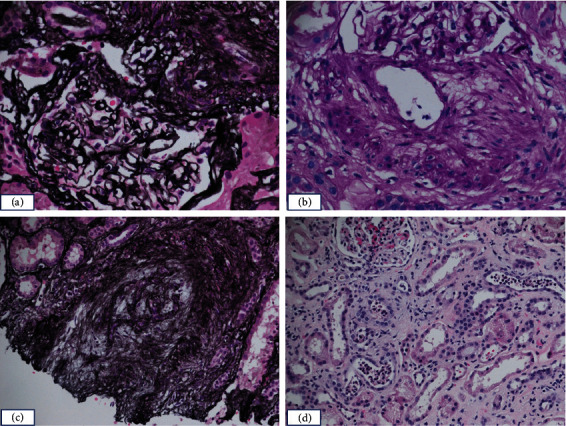
Representative images of histological/pathological findings in a renal biopsy specimen from the patient. (a) Jones silver stain shows a glomerulus in a relatively normal morphology, with no crescents or necrosis noted; adjacent is an arterial with medial hypertrophy. (b) An arterial and PAS stain shows marked intimal thickening/fibrosis. (c) Jones silver stain demonstrates an artery with fibrous obliteration/complete luminal occlusion. (d) H&E stain shows focal intraluminal neutrophilic casts/debris in the tubules, which can be seen in infections or other types of tubular interstitial nephritis. No immune complexes were present in the glomeruli.

The patient had follow-up with nephrology given concern about her abnormal UAs in the setting of suspected MPA. Her creatinine remained slightly elevated at 1.1 with mildly elevated protein/creatinine ratio ranging from 0.17 to 0.90 (normal limit <0.14). Ultimately, the patient underwent a kidney biopsy in mid-August without complications. Biopsy did not show active signs of vasculitis but did have signs that may be representative of previous sequelae of vasculitis. Urine cultures obtained during this time period were all negative. Biopsy results are shown in Figure 1.

She had complete resolution of symptoms noted at follow-up rheumatology visits after the kidney biopsy. The prednisone taper ended the first week of October without any relapse of symptoms. She had serial blood work that demonstrated disease resolution, listed in Table 1, confirming the diagnosis of doxycycline-induced AAV. The patient continues to be seen annually and is doing well. Spring 2020 marked 5 years since her episode of doxycycline-induced AAV.

The following laboratory results are included for reference but will not be discussed in further detail as they did not directly affect the patient's clinical course. Complement levels were normal in July with a mildly decreased complement C4 of 81 noted on mid-August at the time of kidney biopsy. Anti-beta 2 glycoprotein IgA and anti-beta 2 glycoprotein IgM were 103.4 and >150, respectively, at the time of the first admission. Anti-double stranded DNA, ribonucleoprotein antibody, anti-Ro antibody, and anti-La antibody were all negative.

## 3. Discussion

Tetracycline-induced vasculitis is a rare entity that should be explored in a patient presenting with symptoms concerning for the underlying autoimmune process and reporting a recent medication change. Previously, studies have investigated if duration and dosage of the offending drug play an increased role in drug-induced lupus. It was shown that long-term minocycline users had increased risk of drug-induced lupus [14]. It seems likely that drug-induced AAV would follow a similar pattern.

Our patient had an 18-day exposure to doxycycline with systemic symptoms that continued to worsen for several weeks upon discontinuation of the medication. It is not possible to say if her vasculitis would have resolved without treatment of immunosuppressants had the doxycycline been discontinued immediately upon her development of symptoms. She may have had spontaneous resolution of AAV if doxycycline was discontinued at her first sign of symptoms. Fortunately, she had a great response to prednisone with resolution of disease.

A kidney biopsy was not pursued in conjunction with symptoms. Additionally, she had been on oral prednisone 60 mg daily for over 40 days before kidney biopsy was obtained. This is likely why the biopsy did not show evidence of active vasculitis but had findings that were consistent with sequelae of vasculitis. Her UAs never showed dysmorphic RBCs which are specific for glomerulonephritis but not sensitive [15]. Urinalysis resulted in RBCs (highest value 30–49 per high power field) during the period of time when she had symptoms, significantly elevated ANA titer, and MPO antibodies with resolution of RBCs in follow-up UAs when her symptoms were resolved.

An investigation in 2019 by Dam et al. studied neutrophil stimulation from SLE patient's serum and AAV patient's serum, specifically examining the mechanisms of NET formation and composition. The kinetics, induction pathways, and composition of the NETs were distinct in SLE compared to AAV [16]. This study potentially raises the question of whether the particular characteristics of drug-induced AAV and primary AAV may be in part due to intrinsically different NET formation.

This is the first case to be described in literature of doxycycline-induced AAV. It is reported that approximately 5 million prescriptions of doxycycline are made annually, thereby making doxycycline a top 150 most commonly prescribed medication within the United States [17]. We postulate that the rarity of doxycycline-induced autoimmune pathology is due to it having no significant metabolism and no known metabolites found in humans [18, 19]. This is in stark contrast to minocycline which has up to six metabolites reported with numerous confirmed cases of drug-induced lupus and drug-induced AAV [18, 20, 21].

## 4. Conclusion

Tetracycline-induced vasculitis is rare but should be recognized by providers as a potential cause of autoimmune disease symptomatology. The pathogenesis of AAV is still not fully understood, but there is a foundation of knowledge that is continuing to be built upon. Drug-induced AAV patients typically respond well with discontinuation of offending medication; however, they may also need steroids for immunosuppression. Doxycycline is not typically associated with autoimmune pathology likely because it has no known metabolites. Per our literature search, this is the first reported case of doxycycline-induced antineutrophil cytoplasmic antibody vasculitis with the patient having complete remission of disease following a tapered course of prednisone.

## Figures and Tables

**Table 1:** Antinuclear antibodies (ANA), perinuclear-antineutrophil cytoplasmic antibodies (pANCA), and anti-myeloperoxidase antibody (anti-MPO AB).

	Reference range	6/18/15	7/1/15	11/23/15	12/26/15
ANA dilution	Normal < 40 titer	40	2,560	1,280	640
pANCA	Normal titer negative		pANCA positive	pANCA positive	Negative
Anti-MPO AB	Normal < 20.1 units		69.3 units	5.4 units	
CRP	Normal 0–5 mg/L		235 mg/L	10 mg/L	

## References

[1] Jennette J. C., Falk R. J., Bacon P. A. (2013). 2012 revised international chapel hill consensus conference nomenclature of vasculitides. *Arthritis & Rheumatism*.

[2] Jennette J. C., Falk R. J., Hu P., Xiao H. (2013). Pathogenesis of antineutrophil cytoplasmic autoantibody-associated small-vessel vasculitis. *Annual Review of Pathology: Mechanisms of Disease*.

[3] Charles L. A., Falk R. J., Jennette J. C. (1992). Reactivity of antineutrophil cytoplasmic autoantibodies with mononuclear phagocytes. *Journal of Leukocyte Biology*.

[4] Radice A., Sinico R. A. (2005). Antineutrophil cytoplasmic antibodies (ANCA). *Autoimmunity*.

[5] Nakazawa D., Masuda S., Tomaru U., Ishizu A. (2019). Pathogenesis and therapeutic interventions for ANCA-associated vasculitis. *Nature Reviews Rheumatology*.

[6] Jiang X., Khursigara G., Rubin R. (1994). Transformation of lupus-inducing drugs to cytotoxic products by activated neutrophils. *Science*.

[7] Soderberg D., Segelmark M. (2016). Neutrophil extracellular traps in ANCA-associated vasculitis. *Frontiers in Immunology*.

[8] Irizarry-Caro J. A., Carmona-Rivera C., Schwartz D. M., Khaznadar S. S., Kaplan M. J., Grayson P. C. (2018). Brief report: drugs implicated in systemic autoimmunity modulate neutrophil extracellular trap formation. *Arthritis & Rheumatology*.

[9] Bonaci-Nikolic B., Nikolic M. M., Andrejevic S., Zoric S., Bukilica M. (2005). Antineutrophil cytoplasmic antibody (ANCA)-associated autoimmune diseases induced by antithyroid drugs: comparison with idiopathic ANCA vasculitides. *Arthritis Research & Therapy*.

[10] Wiik A. (2005). Clinical and laboratory characteristics of drug-induced vasculitic syndromes. *Arthritis Research & Therapy*.

[11] Chen Y.-X., Zhang W., Chen X.-N. (2012). Propylthiouracil-induced antineutrophil cytoplasmic antibody (ANCA)-associated renal vasculitis versus primary ANCA-associated renal vasculitis: a comparative study. *The Journal of Rheumatology*.

[12] Sloan B., Scheinfeld N. (2008). The use and safety of doxycycline hyclate and other second-generation tetracyclines. *Expert Opinion on Drug Safety*.

[13] Miller K. K., Chu J., Patel R., Kamino H. (2011). Drug-induced subacute cutaneous lupus erythematosus related to doxycycline. *Dermatology Online Journal*.

[14] Sturkenboom M. C. J. M., Meier C. R., Jick H., Stricker B. H. C. (1999). Minocycline and lupuslike syndrome in acne patients. *Archives of Internal Medicine*.

[15] Hamadah A. M., Gharaibeh K., Mara K. C. (2018). Urinalysis for the diagnosis of glomerulonephritis: role of dysmorphic red blood cells. *Nephrology Dialysis Transplantation*.

[16] Dam L. S., Kraaij T., Kamerling S. W. A. (2019). Intrinsically distinct role of neutrophil extracellular trap formation in antineutrophil cytoplasmic antibody-associated vasculitis compared to systemic lupus erythematosus. *Arthritis & Rheumatology*.

[17] Agency for Healthcare Research and Quality Doxycycline drug dosage statistics, United States. https://clincalc.com/DrugStats/Drugs/Doxycycline.

[18] Agwuh K. N., MacGowan A. (2006). Pharmacokinetics and pharmacodynamics of the tetracyclines including glycylcyclines. *Journal of Antimicrobial Chemotherapy*.

[19] Saivin S., Houin G. (1988). Clinical pharmacokinetics of doxycycline and minocycline. *Clinical Pharmacokinetics*.

[20] Elkayam O., Levartovsky D., Brautbar C. (1998). Clinical and immunological study of 7 patients with minocycline-induced autoimmune phenomena. *The American Journal of Medicine*.

[21] Gough A., Chapman S., Wagstaff K., Emery P., Elias E. (1996). Minocycline induced autoimmune hepatitis and systemic lupus erythematosus-like syndrome. *BMJ*.

